# Supplementary material for: Indirect Predictors of Visceral Adipose Tissue in Women with Polycystic Ovary Syndrome: A Comparison of Methods
Source: Nutrients. 2021 Jul 22;13(8):2494. doi: 10.3390/nu13082494 (PMC8401513; doi:10.3390/nu13082494)
Supplement: Supplementary file 1 [file nutrients-13-02494-s001.zip › nutrients-1260486-SI.pdf]

Indirect Predictors of Visceral Adipose Tissue in Women with Polycystic Ovary Syndrome: A Comparison of Methods

Małgorzata Kałużna [1,*], Magdalena Czlapka-Matyasik [2], Aleksandra Bykowska-Derda [2], Jerzy Moczko [3],
Marek Ruchala [1] and Katarzyna Ziemnicka [1]

Table S1. Limits of detection of assessed hormones (electrochemiluminescence (ECLIA), Cobas 6000 equipment, Roche Diagnostics, Switzerland)

Hormone	Limit of detection
AMH (pmol/l)	0.214
DHEAS (µg/dl)	0.100
E2 (pg/ml)	5
FSH (mIU/ml)	0.100
Insulin (µIU/ml)	0.2
LH (mIU/ml)	0.100
SHBG (nmol/l)	0.350
T (nmol/l)	0.087

AMH - *Anti-Müllerian hormone;* DHEAS - *dehydroepiandrosterone sulfate*; E2 - estradiol; FSH - *follicle-stimulating* hormone; LH - *luteinizing hormone*; SHBG - sex hormone *binding* globulin; T - total testosterone.

Table S2. Comparison between study (PCOS) and control (CON) samples age groups (median ± interquartile range [IQR] or percentage).

variable	$PCOS_{18-30}$ n=122	$PCOS_{30-40}$ n=32	P value $PCOS_{18-30}$/ $PCOS_{30-40}$	CON_{18-30} n=49	CON_{30-40} n=19	P value CON_{18-30} /CON_{30-40}	P value PCOS/ CON_{18-30}	P value PCOS/ CON_{30-40}
Age (yrs)	23.8 ± 5.1	32.4 ± 4.6	***	23.3 ± 2.7	33.2 ± 3.9	***	NS	NS
weight (kg)	65.00 ± 20.00	71.50 ± 20.50	NS	62.50 ± 12.50	77.00 ± 10.00	**	NS	NS
BMI (kg/m^2)	23.49 ± 7.44	24.87 ± 7.84	NS	22.01 ± 4.25	28.71 ± 6.06	*	NS	NS
WC (cm)	77.00 ± 19.00	84.00 ± 15.00	NS	72.00 ± 12.00	84.50 ± 14.00	**	NS	NS
WHR (-)	0.88 ± 0.10	0.91 ± 0.12	NS	0.87 ± 0.09	0.92 ± 0.07	*	NS	NS
WHtR (-)	0.46 ± 0.11	0.50 ± 0.13	NS	0.43 ± 0.07	0.50 ± 0.10	NS	NS	NS
WHT.5R (-)	0.59 ± 0.15	0.65 ± 0.14	NS	0.56 ± 0.09	0.65 ± 0.11	**	NS	NS
A/G ratio (-)	0.35 ± 0.21	0.35 ± 0.23	NS	0.32 ± 0.17	0.41 ± 0.20	NS	NS	NS
VAT mass (g)	251.43 ± 521.73	496.96 ± 736.70	NS	174.76 ± 287.00	675.94 ± 426.13	***	NS	NS
TBF (%)	0.35 ± 0.12	0.36 ± 0.11	NS	0.33 ± 0.12	0.37 ± 0.10	NS	NS	NS
FMI (kg/m^2)	8.06 ± 4.44	8.99 ± 5.24	NS	7.55 ± 3.50	10.63 ± 4.84	NS	NS	NS
LAP (-)	13.95 ± 25.68	23.32 ± 47.25	NS	12.00 ± 9.06	29.95 ± 38.48	NS	NS	NS

VAI (-)	0.81 ± 0.85	1.07 ± 1.68	NS	0.91 ± 0.65	1.32 ± 1.17	NS	NS	NS
SBP (mmHg)	120.00 ± 19.00	122.00 ± 21.00	NS	121.00 ± 22.00	125.00 ± 18.00	NS	NS	NS
DBP (mmHg)	74.00 ± 22.00	78.00 ± 8.00	NS	76.00 ± 14.00	76.00 ± 19.00	NS	NS	NS
Glucose (mg/dL)	88.00 ± 8.00	94.00 ± 8.50	***	87.00 ± 11.00	89.50 ± 6.00	NS	NS	NS
Insulin (µU/ml)	9.06 ± 6.78	9.75 ± 8.50	NS	9.38 ± 3.78	10.50 ± 7.41	NS	NS	NS
HOMA-IR	1.96 ± 1.68	2.20 ± 2.21	NS	1.92 ± 0.96	2.04 ± 1.78	NS	NS	NS
TC (mg/dL)	176.00 ± 38.00	186.50 ± 55.00	NS	162.00 ± 42.00	175.50 ± 51.00	NS	NS	NS
TG (mg/dL)	67.00 ± 46.00	90.00 ± 84.00	NS	68.00 ± 48.00	97.00 ± 74.00	NS	NS	NS
HDL-C (mg/dL)	63.00 ± 22.00	62.00 ± 17.00	NS	67.00 ± 27.00	57.50 ± 18.00	NS	NS	NS
LDL-C (mg/dL)	94.90 ± 37.40	99.40 ± 52.20	NS	78.30 ± 29.30	101.65 ± 32.20	NS	NS	NS
TSH (µU/mL)	2.10 ± 1.25	1.73 ± 1.37	NS	2.76 ± 1.81	2.30 ± 1.39	NS	NS	NS
FSH (mIU/mL)	5.80 ± 2.40	6.35 ± 2.50	NS	5.60 ± 4.60	5.50 ± 2.00	NS	NS	NS
LH (mIU/mL)	8.40 ± 8.40	9.60 ± 8.70	NS	6.80 ± 6.80	6.85 ± 2.30	NS	NS	NS

E2 (pg/mL)	42.00 ± 43.00	43.00 ± 30.00	NS	67.50 ± 65.00	68.00 ± 56.00	NS	NS	NS
T (nmol/L)	1.60 ± 1.10	1.65 ± 0.90	NS	1.30 ± 0.90	1.05 ± 1.00	NS	***	***
DHEAS (µg/dL)	321.00 ± 167.00	266.00 ± 166.00	NS	263.00 ± 167.00	275.00 ± 141.00	NS	NS	NS
SHBG (nmol/L)	54.30 ± 32.90	55.45 ± 61.30	NS	55.00 ± 45.10	54.90 ± 20.20	NS	NS	NS
FTI (%)	3.21 ± 3.05	2.99 ± 3.91	NS	2.33 ± 2.49	2.53 ± 2.39	NS	NS	NS
AMH (pmol/L)	53.11 ± 28.35	63.10 ± 41.55	NS	27.74 ± 16.50	14.55 ± 16.38	NS	***	***

Data were presented as median ± interquartile range (IQR) or percentage. AMH - Anti-Müllerian hormone; BMI - body mass index; CON - control subjects; DBP - diastolic blood pressure; DHEAS - dehydroepiandrosterone sulfate; E2 - estradiol; FPG - fasting plasma glucose; FSH - follicle-stimulating hormone; FTI - free testosterone index; LH - luteinising hormone; HDL-C - high-density lipoprotein cholesterol; HOMA-IR - homeostasis model assessment for insulin resistance index; LAP - lipid accumulation product; LDL-C - low-density lipoprotein cholesterol; PCOS - polycystic ovary syndrome patients; SBP - systolic blood pressure; SHBG - sex hormone-binding globulin; T - total testosterone; TC - total cholesterol; TG - triglycerides; VAI - visceral adiposity index; WC - waist circumference; WHtR - waist-to-height ratio; *** for $p < 0.001$, ** for $p < 0.01$, * for $p < 0.05$, NS for not statistically significant.

Table S3. Statistical significance of differences between areas under the curves (AUCs) for visceral adiposity indices in PCOS women (*p* value).

variable	BMI	WC	WHR	WHtR	WHT.5R	A/G ratio	TBF	FMI	LAP	VAI
					PCOS$^{18-30}$					
BMI	-	NS	**	NS	*	**	**	NS	NS	NS
WC	NS	-	***	NS	NS	***	***	**	NS	**
WHR	**	***	-	***	***	NS	NS	NS	***	NS
WHtR	NS	NS	***	-	NS	***	***	**	NS	**
WHT.5R	*	NS	***	NS		***	***	**	NS	**
A/G ratio	**	***	NS	***	***	-	NS	**	***	NS
TBF	**	***	NS	***	***	NS	-	**	***	NS
FMI	NS	**	NS	**	**	**	**	-	*	NS
LAP	NS	NS	***	NS	NS	***	***	*	-	***
VAI	NS	**	NS	**	**	NS	NS	NS	***	-
					PCOS$^{30-40}$					
BMI	-	NS	**	NS	NS	NS	NS	NS	NS	NS
WC	NS	-	**	NS	NS	**	*	NS	NS	NS
WHR	**	**	-	**	**	NS	NS	*	**	*
WHtR	NS	NS	**	-	NS	***	*	NS	NS	NS
WHT.5R	NS	NS	**	NS	-	**	*	NS	NS	NS

A/G ratio	NS	**	NS	***	**	-	NS	NS	*	NS
TBF	NS	*	NS	*	*	NS	-	*	NS	NS
FMI	NS	NS	*	NS	NS	NS	*	-	NS	NS
LAP	NS	NS	**	NS	NS	*	NS	NS	-	NS
VAI	NS	NS	*	NS	NS	NS	NS	NS	NS	-

A/G ratio – android-to-gynoid ratio; BMI - body mass index; CON - control subjects; FMI - fat mass index, LAP - lipid accumulation product; PCOS - polycystic ovary syndrome patients; TBF - total body fat percentage, VAI - visceral adiposity index; VAT - visceral adipose tissue mass; WC - waist circumference; WHR - waist-to-hip ratio; WHtR - waist-to-height ratio. *** for $p < 0.001$, ** for $p < 0.01$, * for $p < 0.05$, ns for not statistically significant.

Table S4. Statistical significance of differences between areas under the curves (AUCs) for selected visceral adiposity indices in CON women (*p* value).

**variable**	***BMI***	***WC***	**WHR**	**WHtR**	***WHT.5R***	***A/G ratio***	***TBF***	***FMI***	***LAP***	***VAI***
					CON[18-30]					
BMI	-	NS	0.003	NS	0.01	NS	NS	NS	NS	NS
WC	NS	-	0.002	NS	NS	NS	NS	NS	NS	NS
WHR	0.003	0.002	-	0.014	0.004	NS	NS	0.03	0.04	NS
WHtR	NS	NS	0.014	-	NS	NS	NS	NS	NS	NS
WHT.5R	0.01	NS	0.004	NS	-	NS	NS	NS	NS	NS

A/G ratio	NS	NS	NS	NS	NS	-	NS	NS	NS	NS
TBF	NS	NS	NS	NS	NS	NS	-	NS	NS	NS
FMI	NS	NS	0.03	NS	NS	NS	NS	-	NS	NS
LAP	NS	NS	0.04	NS	NS	NS	NS	NS	-	NS
VAI	NS	NS	NS	NS	NS	NS	NS	NS	NS	-
CON $^{30-40}$										
BMI	-	<0.001	<0.001	0.002	<0.001	<0.001	0.002	NS	<0.001	<0.001
WC	<0.001	-	<0.001	NS	NS	NS	NS	NS	0.013	0.03
WHR	<0.001	<0.001	-	NS	0.001	NS	NS	NS	NS	NS
WHtR	0.002	NS	NS	-	NS	NS	NS	NS	NS	0.002
WHT.5 R	<0.001	NS	0.001	NS	-	NS	NS	0.006	0.008	0.03
A/G ratio	<0.001	NS	NS	NS	NS	-	NS	<0.001	NS	NS
TBF	0.002	NS	NS	NS	NS	NS	-	<0.001	NS	NS
FMI	NS	NS	0.008	NS	0.006	<0.001	<0.001	-	0.03	0.02
LAP	<0.001	0.013	NS	NS	0.008	NS	NS	0.03	-	NS
VAI	NS	0.03	NS	0.002	0.03	NS	NS	NS	NS	-

A/G ratio – android-to-gynoid ratio; BMI - body mass index; CON - control subjects; FMI - fat mass index, LAP - lipid accumulation product; PCOS - polycystic ovary syndrome patients; TBF - total body fat percentage, VAI - visceral adiposity index; VAT - visceral adipose tissue mass; WC - waist circumference; WHR - waist-to-hip ratio; WHtR - waist-to-height ratio.
